# Case series: Diquat poisoning with acute kidney failure, myocardial damage, and rhabdomyolysis

**DOI:** 10.3389/fpubh.2022.991587

**Published:** 2022-10-24

**Authors:** Guangcai Yu, Jieru Wang, Tianzi Jian, Longke Shi, Liwen Zhao, Yaqian Li, Yikai Gao, Baotian Kan, Xiangdong Jian

**Affiliations:** ^1^Department of Poisoning and Occupational Diseases, Emergency Medicine, Qilu Hospital of Shandong University, Cheeloo College of Medicine, Shandong University, Jinan, China; ^2^Shandong University of Traditional Chinese Medicine, Jinan, China; ^3^Department of Critical Care Medicine, The 5th People's Hospital of Jinan, Jinan, China; ^4^School of Public Health, Cheeloo College of Medicine, Shandong University, Jinan, China; ^5^Department of Critical Care Medicine, Shandong Provincial Third Hospital, Shandong University, Jinan, China; ^6^School of Nursing and Rehabilitation, Cheeloo College of Medicine, Shandong University, Jinan, China

**Keywords:** diquat poisoning, acute kidney injury, rhabdomyolysis, myohemoglobin, hemopurification

## Abstract

Diquat is a herbicide that can have deleterious effects on the kidneys, liver, heart, lungs, and central nervous system on ingestion. Diquat poisoning-associated rhabdomyolysis has rarely been reported. We describe two cases of diquat poisoning with acute renal failure, myocardial damage, and rhabdomyolysis. Case 1: A 17-year-old man experienced anuria after ingesting ~200 mL of diquat 16 h prior. On admission, his creatinine (400 μmol/L), urea (11.7 mmol/L), creatine kinase (2,534 IU/L), and myohemoglobin (4,425 ng/mL) concentrations were elevated. Case 2: An 18-year-old woman who ingested ~200 mL of diquat 5.5 h prior to admission had normal creatinine, urea, and creatine kinase concentrations. Eleven hours after ingestion, she developed anuria with elevated creatinine (169 μmol/L) concentration; her creatine kinase (13,617 IU/L) and myohemoglobin (>3,811 ng/mL) concentrations were remarkably elevated 24 h after ingestion. Both patients also had elevated aminotransferase and myocardial enzyme concentrations. After undergoing hemoperfusion and hemofiltration, blood diquat concentrations in cases 1 and 2 on admission (16/6 h after ingestion), after hemoperfusion (20/11 h after ingestion), and after 8 h of hemofiltration/8 h of hemofiltration and 2 h of hemoperfusion (29/21 h after ingestion) were 4.9/9.1, 3.4/5.4, and 1.5/1.2 μg/mL, respectively. Severe diquat poisoning can cause acute kidney failure and rhabdomyolysis. Rhabdomyolysis may induce myocardial injury, aggravating kidney damage, and also increase transaminase concentration. Hemoperfusion and hemofiltration could be effective treatments for eliminating diquat in the blood.

## Introduction

Diquat is a non-selective contact herbicide used for agricultural weed control (1). Diquat toxicity occurs through the production of oxygen free radicals, causing oxidative stress and leading to cell death and multiple organ dysfunction syndrome, commonly affecting the kidneys, liver, heart, lungs, and central nervous system (1–3). In the kidneys, diquat causes tubular necrosis, oliguria, and anuria, which are the main causes of death (1, 3). Rhabdomyolysis also leads to acute kidney injury (4). Only a few reported cases of diquat poisoning demonstrated elevated creatine kinase (CK) and myoglobin (MYO) levels (5–7), but the clinical implications of rhabdomyolysis have not been sufficiently described. We report two cases of diquat poisoning with rhabdomyolysis-related myocardial injury and elevated transaminases, highlighting the challenges imposed by rhabdomyolysis in acute renal failure and myocardial damage. Hemoperfusion and/or hemofiltration for reducing blood diquat concentration is another highlight.

## Case presentation

### Case 1

**Figure 1:**
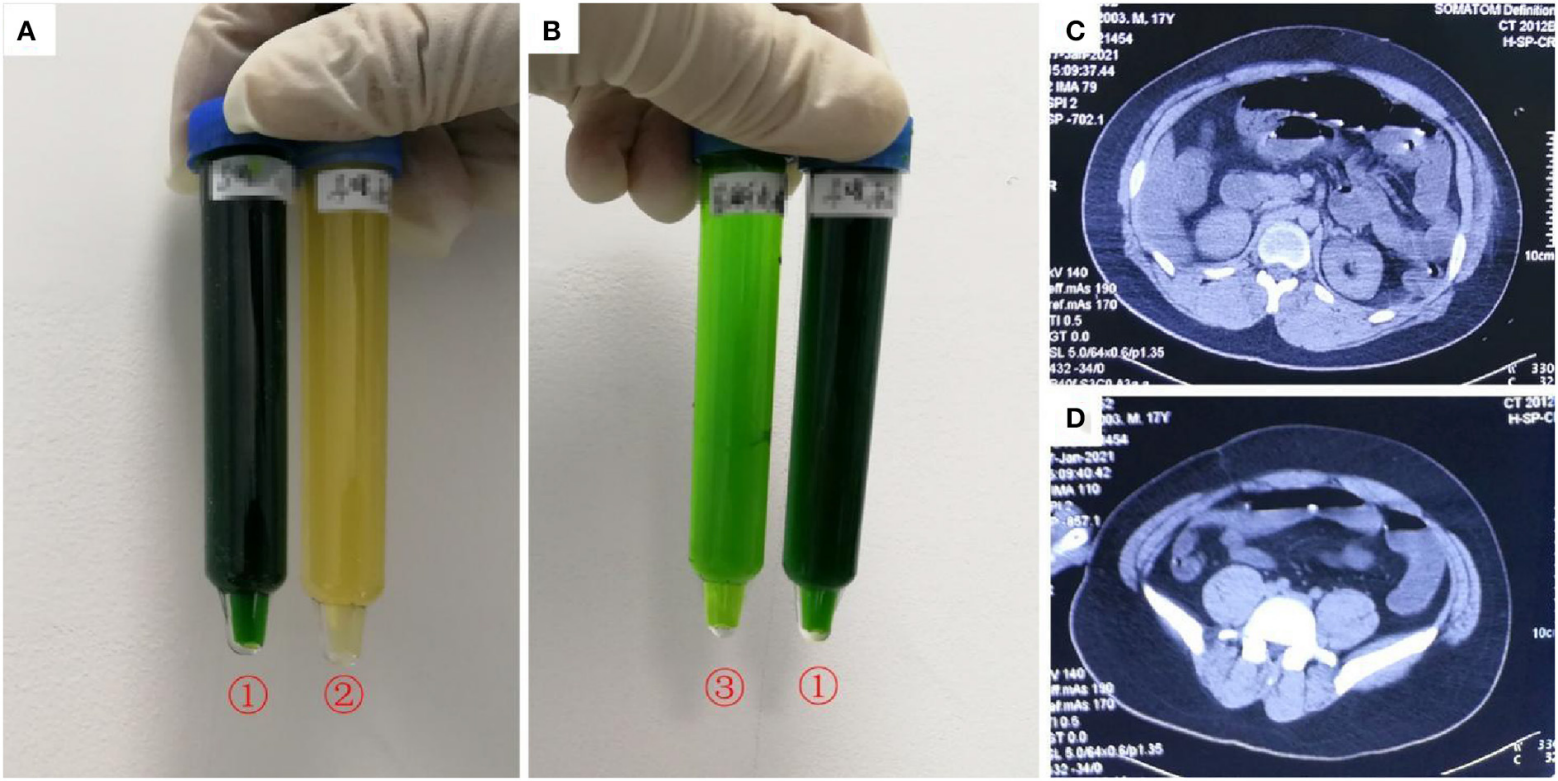
Images of urine and computerized tomography (CT) for the 21-year-old man who ingested ~200 mL of diquat. As shown in **(A,B)**, after exposure to sodium dithionite, the patient's urine turned dark green (tube no. 1); the patient's original urine was darker in color (tube no. 2); the patient's urine turned light green after dilution (tube no. 3). **(C,D)** Shows partial intestinal gas and dilatation at the fluid level.

A 17-year-old previously healthy boy (height: 170 cm, weight: 65 kg) visited a local hospital 1 h after ingesting ~200 mL diquat (20 g/100 mL). Gastric lavage and 2 h of hemoperfusion were performed. Methylprednisolone (200 mg), pantoprazole (40 mg twice a day), reduced glutathione (2.4 g), furosemide (20 mg, intravenous, twice a day), and mannitol were administered as cathartics. Nine hours after ingestion, he developed restlessness and oliguria. Sixteen hours after ingestion, he was transferred to our department during which he developed chest tightness. Physical examination revealed impaired consciousness, with a Glasgow coma scale score of 12 (E4V3M5). His vital signs were as follows: heart rate, 168 beats/min; blood pressure, 167/108 mmHg; respiratory rate, 42 breaths/min, and oxygen saturation, 97%. Physical examination revealed no other abnormalities despite abdominal distention and weakening of abdominal sounds; his urine color based on the sodium bicarbonate/dithionite test was dark green (Figures 1A,B). His blood diquat concentration was 4.9 μg/mL. His creatinine (400 μmol/L), urea (11.7 mmol/L), and alanine aminotransferase (ALT) and aspartate aminotransferase (AST) concentrations were elevated, but his total bilirubin (TBIL) concentration was normal; his CK and myohemoglobin (MYO) concentrations were 2,534 IU/L and 4,425 ng/mL, respectively. His blood β2-microglobulin (12.7 mg/L, normal value 0.7–1.8 mg/L), urine β_2_-microglobulin (165 mg/L, normal value <0.22 mg/L), and urine microalbumin (3,040 mg/L, normal value <30 mg/L) concentrations were elevated. Computed tomography (CT) revealed partial intestinal gas and dilatation at the fluid level (Figures 1C,D) as well as diffuse cerebral edema. Endotracheal intubation and hemoperfusion (HA330, Jafron, China, 2 h/treatment) were performed. Treatment included midazolam, disoprofol, esmolol, betamethasone (8 mg), pantoprazole (40 mg, twice a day), reduced glutathione (2.4 g), alanyl glutamine (20 g), and furosemide (80 mg twice a day). Twenty hours after ingestion, the patient was anuric after hemoperfusion; his blood diquat concentration was 3.4 μg/mL. Continuous veno-venous hemofiltration (CVVH; Prismaflex ST100, Gambro, Sweden) was immediately performed. After 8 h of CVVH, his blood diquat concentration decreased to 1.5 μg/mL. The patient experienced progressive anuria and abdominal distension. Repeated hemoperfusion and subsequent continuous veno-venous hemodiafiltration (CVVHDF; Prismaflex ST100) were performed. The patient then developed respiratory failure caused by pulmonary edema, and mechanical ventilation was performed. The patient died 40.5 h after diquat ingestion owing to refractory circulatory collapse and progressive hypoxemia. The patient's main laboratory tests results were shown in Table 1 (Case 1).

**Table 1:** Patients' laboratory test results.

**Inspection items**	**Normal range**	**Case 1**			**Case 2**		
		**At 16 h after ingestion**	**At 20 h after ingestion (after first HP)**	**At 29 h after ingestion^#^**	**At 6 h after ingestion**	**At 11 h after ingestion (after first HP)**	**At 21 h after ingestion^#^**
WBC count (×10^9^/L)	3.5–9.5	21.5	N	22.0	28.5	N	41.6
NEU %	40–75	92	N	93.6	93.8	N	97.2
Hemoglobin (g/L)	115–150	187	N	179	137	N	148
Platelet (×10^9^/L)	125–350	187	N	151	390	N	270
ALT (IU/L)	9–52	1,017	N	894	19	N	1,112
AST (IU/L)	14–36	721	N	528	44	N	1,576
TBIL (μmol/L)	3–22	18	N	17	20	N	14
GGT (IU/L)	15–73	53	N	47.5	12	12	20
Creatinine (μmol/L)	46–106	400	411	478	105	169	229
Urea (mmol/L)	2.5–6.1	11.7	12.7	15.1	3.9	5.5	9.7
LDH (IU/L)	313–618	9,728	10,254	8,803	653	3,603	N
CK (IU/L)	30–135	2,534	2,419	1,437	177	13,617	52,001
CK-MB (ng/mL)	0.3–4	23.8	31	25	7.4	115	449
CTNI (ng/mL)	<30	124	153.1	72.33	N	3.49	N
MYO (ng/mL)	0–70	4,426	4,697	2,914	3,223.1	>3,811	44,192.2
NT-PRO-BNP (pg/mL)		2,113	N	4,002	N	2,463	N
K^+^ (mmol/L)	3.5–5.5	4.63	4.71	3.79	3.8	3.7	4.8
Na^+^ (mmol/L)	135–145	140	140.3	135.7	146	138	130
DQ (μg/mL)	<0.1	4.9	3.4	1.5	9.1	5.4	1.2

HP, hemoperfusion; WBC, white blood cell; NEU, neutrophil; ALT, alanine aminotransferase; AST, aspartate aminotransferase; TBIL, total bilirubin; GGT, gamma-glutamyl transpeptidase; LDH, lactate dehydrogenase; CK, creatine kinase; CK-MB, creatine kinase-MB; CTNI, cardiac troponin I; MYO, myohemoglobin; NT-PRO-BNP, N-terminal pro-Brain Natriuretic Peptide; K^+^, serum potassium; Na^+^, serum sodium; DQ, blood diquat concentration; N, not examined.

^#^Case 1 was after 8 h of hemofiltration; Case 2 was after 8 h of hemofiltration and 2 h of hemoperfusion.

### Case 2

**Figure 2:**
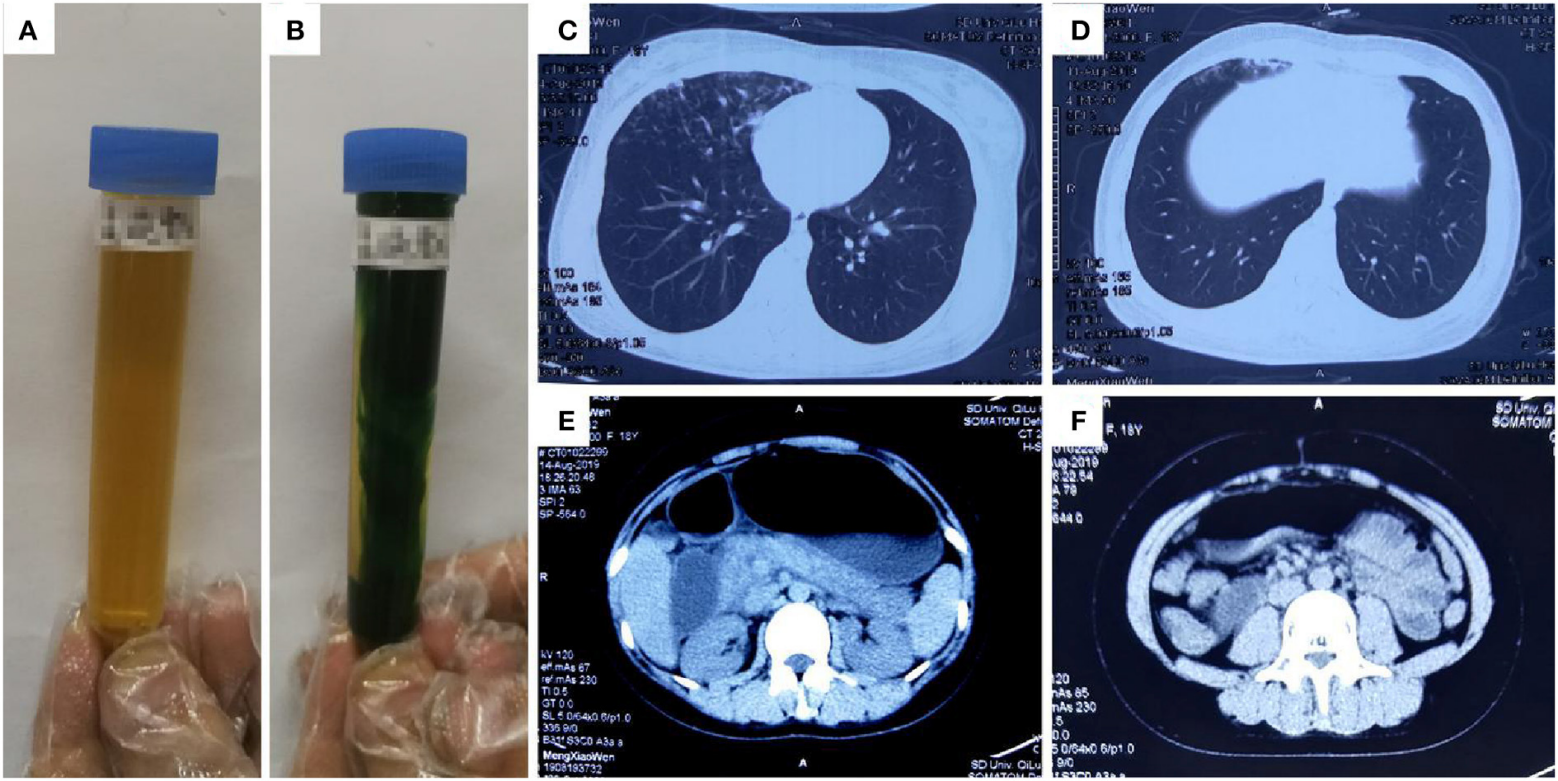
Images of urine and computerized tomography (CT) for the 18-year-old woman who ingested ~200 mL of diquat. As shown in **(A)**, the patient's original urine was dark brown in color: after exposure to sodium dithionite, the patient's urine turned dark green **(B)**. The images were taken 11 h after ingestion; **(C,D)** show lung damage, and **(E,F)** show gastrointestinal gas, dilatation, and fluid level; The images were taken 11 h after ingestion.

An 18-year-old previously healthy woman (height: 162 cm, weight: 57 kg) attempted suicide by ingesting ~200 mL diquat (20 g/100 mL). Gastric lavage was performed. She was transferred to our department 5.5 h after ingestion. On admission, she was conscious and reported a burning sensation throughout her entire body. Her physical examination results were normal, except for tachycardia (111 beats/min). Her blood diquat concentration was 9.1 μg/mL; her liver and renal functions were normal. Hemoperfusion (HA330) was performed. She received intravenous medical treatment similar to that in Case 1. She also received treatment *via* gastrointestinal adsorption and catharsis using smectite powder and activated charcoal with mannitol taken orally. Eleven hours after ingestion, she developed dysphoria, anuria, abdominal distension, and weakening of abdominal sounds. Her blood diquat concentration reduced to 5.4 μg/mL, while CK rose from 177 IU/L (on admission) to 13,617 IU/L; her MYO increased significantly, but her creatinine (169 μmol/L) and urea (5.5 mmol/L) concentrations were mildly elevated. CT showed partial lung interstitial infiltration, gastrointestinal gas, and dilatation with fluid levels (Figure 2). Hemoperfusion (2 h) and CVVH were performed. Twenty-one hours after ingestion, she became comatose and had anuria and pink foam sputum. Her blood diquat concentration reduced to 1.2 μg/mL, but her CK (52,001 IU/L) and MYO (44,192.2 ng/mL) concentrations increased further. Electrocardiography showed sinus tachycardia (165 beats/min). The patient died of cardiac arrest 29 h after ingestion. The patient's main laboratory tests results were shown in Table 1 (Case 2).

### Ethical approval

Ethical approval was obtained from the Qilu Hospital Ethics Committee for Human Research, and written informed consent was obtained from patients' families.

## Discussion

For severe diquat poisoning cases, multiple organ injuries can occur within a few hours after ingestion, and the clinical manifestations and prognosis are mainly related to the dose of the poison. Zhou and Lu (8) observed that the high mortality in diquat poisoning cases is primarily associated with serious brain and vascular injury. Consciousness disorder, paralytic ileus, myocardial damage, acute kidney failure, and rhabdomyolysis were important clinical findings in our cases. Diquat-related toxic encephalopathy (9) and paralytic ileus (10) were reported in our previous studies. Patients with severe cases may develop acute kidney injury, as noted in the present cases, as diquat accumulates in high concentrations in the kidneys (1). Animal studies demonstrate that diquat can easily cause edema, degeneration, and necrosis of the renal tubules (11). Major pathological changes observed on autopsy include congested kidneys, necrotic renal tubules, and intact glomeruli (12, 13). In Case 1, the patient's urine β2-microglobulin concentration increased significantly, suggesting severe damage of the proximal convoluted tubule. His blood β2-microglobulin concentration increased, which was likely secondary to severe renal impairment. Rhabdomyolysis is a potentially life-threatening condition characterized by skeletal muscle breakdown and leakage of intracellular substances into the circulation (14). In this paper, both patients' CK and MYO concentrations increased remarkably, indicating muscle damage and rhabdomyolysis; the increase can, in turn, aggravate acute kidney injury. Before discovery of rhabdomyolysis, both patients were not comatose, convulsing, or co-exposed to any other toxins. Therefore, the underlying mechanisms of rhabdomyolysis may be related to mitochondrial damage caused by oxyradicals and cell membrane damage caused by lipid peroxidation, which led to cellular injury or death (15).

In this case report, we also found that the two patients' ALT, AST, MYO, CK, CK-MB, and cardiac troponin I concentrations were elevated without an increase in gamma-glutamyl transpeptidase and bilirubin levels. Elevated transaminase levels (ALT and AST) are common clinical characteristics of acute diquat poisoning. Jović-Stosić et al. (5) reported on a 35-year-old woman with significant elevation of transaminase levels after ingesting diquat, but her liver only showed congestion at autopsy. Wu et al. (11) reported acute tubular injury and minimal pathological findings in the liver of diquat-intoxicated rats. Jones et al. (16) had hypothesized that diquat-induced liver injury is typically mild, transient, and resolves spontaneously. We speculated that the elevated concentrations of aminotransferases may be partly associated with skeletal and cardiac muscle injuries. Ababou et al. (17) reported the case of a 20-year-old woman who ingested 4 g of paraphenylene diamine and experienced lethal cardiogenic shock, which was confirmed to be secondary to myocardial rhabdomyolysis in a post-mortem biopsy. Herein, we assumed that myocardial injury was associated with diquat-induced myocardial rhabdomyolysis, which was the leading cause of refractory circulatory failure and even cardiac arrest. Moreover, acute renal failure can further aggravate myocardial damage.

The blood diquat concentration can be reduced *via* liver and kidney metabolisms, and through stomach lavage, vomiting, and laxative use. Owing to the lack of a specific antidote, it is necessary to reduce absorption and enhance elimination in oral diquat poisoning (1). Hemoperfusion is a common method for removing toxins from the blood, and hemofiltration is used for treating acute renal failure and rhabdomyolysis (18, 19). In this case report, diquat concentration in the blood was reduced *via* hemoperfusion and hemofiltration. Previous studies reported a higher concentration of diquat in the liver, brain, kidney, and skeletal muscles than in the blood in the early stages of poisoning (1, 11, 16). Thus, we hypothesized that hemoperfusion and hemofiltration eliminate poisons in tissues at a lower rate than that in blood. In addition, our cases did not show a decrease in serum creatinine and CK concentrations, which might be related to the large quantities of poison in tissue cells and the resulting tissue and organ damage. In this study, both patients underwent timely stomach lavage, but they had early stage paralytic ileus, which was unfavorable for removal of diquat from the intestinal tract. Therefore, we assumed that timely and proper blood purification might be beneficial for severe cases of diquat poisoning.

## Data availability statement

The raw data supporting the conclusions of this article will be made available by the authors, without undue reservation.

## Ethics statement

The studies involving human participants were reviewed and approved by Qilu Hospital Ethics Committee for Human Research. The patients/participants provided their written informed consent to participate in this study.

## Author contributions

GY conceived the study, drafted the manuscript, and takes responsibility for the paper as a whole. GY, JW, YL, YG, and LS supervised the conduct of the paper and data collection. GY, JW, TJ, and YL provided statistical advice on study design and analyzed the data. BK and XJ chaired the data oversight committee. All authors contributed substantially to manuscript revision. All authors contributed to the article and approved the submitted version.

## Funding

This work was supported by the Qilu Hospital, Shandong University under Grant KYLL-2019-296.

## Conflict of interest

The authors declare that the research was conducted in the absence of any commercial or financial relationships that could be construed as a potential conflict of interest.

## Publisher's note

All claims expressed in this article are solely those of the authors and do not necessarily represent those of their affiliated organizations, or those of the publisher, the editors and the reviewers. Any product that may be evaluated in this article, or claim that may be made by its manufacturer, is not guaranteed or endorsed by the publisher.

## References

[1] Magalhães N Carvalho F Dinis-Oliveira RJ. Human and experimental toxicology of diquat poisoning: toxicokinetics, mechanisms of toxicity, clinical features, and treatment. Hum Exp Toxicol. (2018) 37:1131–60. 10.1177/0960327118765330 29569487

[2] Ness-Cochinwala M Proaño JS Bernstein JN Martinez P Ladd H Totapally B. case of a lethal diquat ingestion in a toddler. J Emerg Med. (2022) 62:e16–9. 10.1016/j.jemermed.2021.10.007 34836733

[3] Xing J Chu Z Han D Jiang X Zang X Liu Y. Lethal diquat poisoning manifesting as central pontine myelinolysis and acute kidney injury: a case report and literature review. J Int Med Res. (2020) 48:300060520943824. 10.1177/0300060520943824 32734801 PMC7401049

[4] Lim AKH Azraai M Pham JH Looi WF Bennett C. The association between illicit drug use and the duration of renal replacement therapy in patients with acute kidney injury from severe rhabdomyolysis. Front Med. (2020) 7:588114. 10.3389/fmed.2020.588114 33240909 PMC7680872

[5] Jović-Stosić J Babić G Todorović V. Fatal diquat intoxication. Vojnosanit Pregl. (2009) 66:477–81. 10.2298/VSP0906477J 19583147

[6] Basilicata P Pieri M Simonelli A Capasso E Casella C Noto T. Diquat poisoning: care management and medico-legal implications. Toxics. (2022) 10:166. 10.3390/toxics10040166 35448427 PMC9030962

[7] Feng D Fu L Du X Yao L. Acute diquat poisoning causes rhabdomyolysis. Am J Med Sci. (2022) 364:472–80. 10.1016/j.amjms.2022.04.032 35508282

[8] Zhou JN Lu YQ. Lethal diquat poisoning manifests as acute central nervous system injury and circulatory failure: a retrospective cohort study of 50 cases. EClinicalMedicine. (2022) 52:101609. 10.1016/j.eclinm.2022.101609 35990582 PMC9386369

[9] Yu G Jian T Cui S Shi L Kan B Jian X. Acute diquat poisoning resulting in toxic encephalopathy: a report of three cases. Clin Toxicol. (2022) 60:647–50. 10.1080/15563650.2021.2013495 34982016

[10] Yu G Cui S Jian T Kan B Jian X. Diquat poisoning in a pregnant woman resulting in a miscarriage and maternal death. Clin Toxicol. (2021) 59:1275–7. 10.1080/15563650.2021.1905164 33853459

[11] Wu Y Cui S Wang W Jian T Kan B Jian X. Kidney and lung injury in rats following acute diquat exposure. Exp Ther Med. (2022) 23:275. 10.3892/etm.2022.11201 35251341 PMC8892614

[12] Vanholder R Colardyn F De Reuck J Praet M Lameire N Ringoir S. Diquat intoxication: report of two cases and review of the literature. Am J Med. (1981) 70:1267–71. 10.1016/0002-9343(81)90836-6 7015857

[13] Hantson P Wallemacq P Mahieu P. A case of fatal diquat poisoning: toxicokinetic data and autopsy findings. J Toxicol Clin Toxicol. (2000) 38:149–52. 10.1081/CLT-100100930 10778912

[14] Vangstad M Bjornaas MA Jacobsen D. Rhabdomyolysis: a 10-year retrospective study of patients treated in a medical department. Eur J Emerg Med. (2019) 26:199–204. 10.1097/MEJ.0000000000000510 29068810

[15] Choi SE Park YS Koh HC. NF-κB/p53-activated inflammatory response involves in diquat-induced mitochondrial dysfunction and apoptosis. Environ Toxicol. (2018) 33:1005–18. 10.1002/tox.22552 29484840

[16] Jones GM Vale JA. Mechanisms of toxicity, clinical features, and management of diquat poisoning: a review. J Toxicol Clin Toxicol. (2000) 38:123–8. 10.1081/CLT-100100926 10778908

[17] Ababou A Ababou K Mosadik A Lazreq C Sbihi A. Myocardial rhabdomyolysis following paraphenylene diamine poisoning. Ann Fr Anesth Reanim. (2000) 19:105–7. 10.1016/S0750-7658(00)00113-1 10730172

[18] Xiao Q Wang W Qi H Gao X Zhu B Li J. Continuous hemoperfusion relieves pulmonary fibrosis in patients with acute mild and moderate paraquat poisoning. J Toxicol Sci. (2020) 45:611–7. 10.2131/jts.45.611 33012729

[19] Sun H Mao Z Mei Y Chen F Zhang J. Case report of intramuscular injection of diquat. Clin Toxicol. (2022) 60:986–8. 10.1080/15563650.2022.2058530 35384770

